# Supplementary material for: The Diagnostic Significance of SLC26A2 and Its Potential Role in Ulcerative Colitis
Source: Biomedicines. 2025 Feb 13;13(2):461. doi: 10.3390/biomedicines13020461 (PMC11853232; doi:10.3390/biomedicines13020461)
Supplement: Supplementary file 1 [file biomedicines-13-00461-s001.zip › biomedicines-3361333-supplementary.pdf]

biomedicines

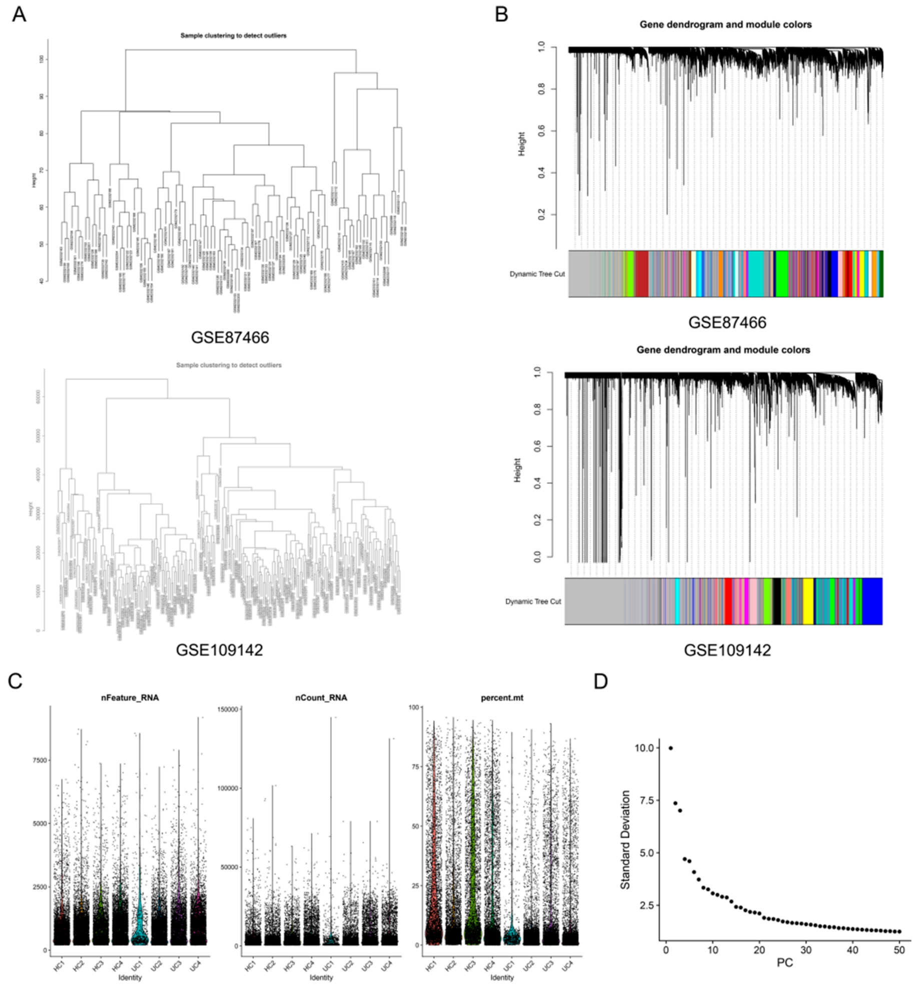

Figure S1. Sample clusters and quality control. (A) The cluster tree showed the clusters of samples after removing the outliers. (B) The dendrogram displayed the different modules in which genes were clustered. (C) The violin plot presented the gene count, mRNA count, and the ratio of mitochondrion in each cell in single-cell RNA sequencing (scRNA-seq) data. (D) The elbow plot indicated the relationship between the count of principal components and standard deviation.
